# Supplementary material for: Natural Products Are a Promising Source for Anthelmintic Drug Discovery
Source: Biomolecules. 2021 Oct 4;11(10):1457. doi: 10.3390/biom11101457 (PMC8533416; doi:10.3390/biom11101457)
Supplement: Supplementary file 1 [file biomolecules-11-01457-s001.zip › biomolecules-1357273-supplementary.pdf]

Supplementary Table S1. Traditional medicinal plant extracts evaluated in vitro and in vivo for anthelmintic activity from year 2000 to 2019.

**Plant Species**	**Model System**	**In Vitro/In Vivo**	**Reference**
Tulbaghia violacea	*Caenorhabditis elegans*	in vitro	[1]
Rhus geinzii	*Schistosoma*	in vitro	[2]
Piliostigma thonningii	*Schistosoma*	in vitro	[3]
Ocimum gratissimum	*Schistosoma*	in vitro	[3]
Nauclea latifolia and	*Schistosoma*	in vitro	[3]
Alstonia boonei	*Schistosoma*	in vitro	[3]
Buchholzia coriaceae	*Pheretima posthuma*	in vitro	[4]
Gynandropsis gynandra	*Pheretima posthuma*	in vitro	[4]
Combretum apiculatum	*Caenorhabditis elegans*	in vitro	[5]
Berlina grandiflora	*Caenorhabditis elegans*	in vitro	[6]
Abrus precatorius	*Schistosoma mansoni*	in vitro	[7]
Elephantorrhiza goetzei	*Schistosoma mansoni*	in vitro	[7]
Evolvulus alsinoides	*Pheritima posthuma*	in vitro	[8]
Myrsine africana	*Haemonchus contortus*-Sheep model	in vitro	[9]
Rapanea melanophloeos	*Haemonchus contortus*- Sheep model	in vitro	[9]
Vernonia amygdalina (leaf)	*Haemonchus contortus*	in vitro	[10,11]
Annona senegalensis	*Haemonchus contortus*	in vitro	[10]
Aframomum sanguineum,	*Heligmosomoides polygyrus* in mouse model	in vitro	[12]
Dodonea angustifolia	*Heligmosomoides polygyrus* in mouse model	in vitro	[12]
Hildebrandtia sepalosa,	*Heligmosomoides polygyrus* in mouse model	in vitro	[12]
Myrsine africana	*Heligmosomoides polygyrus* in mouse model	in vitro	[12]
Rapanea melanophloeos	*Heligmosomoides polygyrus* in mouse model	in vitro	[12]

Azadirachta indica.	*Heligmosomoides polygyrus* in mouse model	in vitro	[12]
Khaya senegalensis	*Strongyles Spp Caenorhabditis elegans Onchocerca ochengi Echinostoma caproni*	in vitro	[13-15]
Artemisia brevifolia	*Haemonchus contortus* Gastrointestinal nematodes- Sheep model	in vitro and in vivo	[16]
Gossypium herbaceum	*Caenorhabditis elegans*	in vitro	[17]
Hertia pallens	*Caenorhabditis elegans*	in vitro	[17]
Jatropha multifida	*Caenorhabditis elegans*	in vitro	[17]
Lantana rugosa	*Caenorhabditis elegans*	in vitro	[17]
Onobrychis viciifolia	*Haemonchus contortus*	in vitro	[18]
leucaena leucocephala	*Haemonchus contortus*	in vitro	[19]
Echinops ellenbeckii	Earth worm	in vitro	[20]
Echinops longisetus	Earth worm	in vitro	[20]
Cannabis sativa	*Ascaris suum*	in vitro	[21]
Moringa oleifera	*Ascaris suum*	in vitro	[21]
Carica papaya	*Ascaris suum*	in vitro	[21]
Cassia occidentalis	*Ascaris suum*	in vitro	[21]
Momordica foetida	*Ascaris suum*	in vitro	[21]
Erythrina abyssinica	*Ascaris suum*	in vitro	[21]
Tetradenia ripara	*Ascaris suum*	in vitro	[21]
Peltophorum africanum	*Trichostrongylus colubriformis*	in vitro	[22]
Zingiber officinale Roscoe	Sheep model with mix gastrointestinal parasites	in vivo	[23]
Coriandrum sativum	*Haemonchus contortus* *H. contortus* with sheep model	in vitro and in vivo	[24]
Sapium sebiferum	*Bursaphelenchus xylophilus, Panagrellus redivivus*	in vitro	[25]
Magnolia grandiflora	*Bursaphelenchus xylophilus, Panagrellus redivivus*	in vitro	[25]

Michelia hedyosperma	*Bursaphelenchus xylophilus, Panagrellus redivivus*	in vitro	[25]
Punica granatum	*Bursaphelenchus xylophilus, Panagrellus redivivus*	in vitro	[25]
Zingiber striolatum	*Bursaphelenchus xylophilus, Panagrellus redivivus*	in vitro	[25]
Edgeworthia chrysantha	*Bursaphelenchus xylophilus, Panagrellus redivivus*	in vitro	[25]
Nerium indicum	*Bursaphelenchus xylophilus, Panagrellus redivivus*	in vitro	[25]
Pongamia glabra	*Pheretima posthuma*	in vitro	[26]
Cissus quadrangularis	*Caenorhabditis elegans*	in vitro	[27]
Pouzolzia mixta Solms	*Caenorhabditis elegans*	in vitro	[27]
Gnidia capitata	*Caenorhabditis elegans*	in vitro	[27]
Dombeya rotundifolia	*Caenorhabditis elegans*	in vitro	[27]
Hippobromus pauciflorus	*Caenorhabditis elegans*	in vitro	[27]
Ziziphus mucronata	*Caenorhabditis elegans*	in vitro	[27]
Berchemia zeyheri	*Caenorhabditis elegans*	in vitro	[27]
Dicerocaryum eriocarpum	*Caenorhabditis elegans*	in vitro	[27]
Schotia brachypetala	*Caenorhabditis elegans*	in vitro	[27]
Pterocarpus angolensis	*Caenorhabditis elegans*	in vitro	[27]
Synadenium cupulare	*Caenorhabditis elegans*	in vitro	[27]
Ricinus communis	*Caenorhabditis elegans*	in vitro	[27]
Schkuhria pinnata	*Caenorhabditis elegans*	in vitro	[27]
Cussonia spicata	*Caenorhabditis elegans*	in vitro	[27]
Secamone filiformis	*Caenorhabditis elegans*	in vitro	[27]
Sclerocarya birrea	*Caenorhabditis elegans*	in vitro	[27]
Rhus lancea	*Caenorhabditis elegans*	in vitro	[27]
Caesalpinia crista	*H. contortus*	in vitro and in vivo	[28]
Chenopodium album	*H. contortus*	in vitro and in vivo	[28]
Terminalia chebula(fruit)	*Pheretima posthuma*	in vitro	[29]
Terminalia chebula(Seed)	*Haemonchus contortus*	in vitro	[30]

Azadirachta indica	*Haemonchus contortus*	in vitro	[31]
Ficus benghalensis	*Pheretima posthuma*	in vitro	[32,33]
Curcuma longa	*Schistosoma mansoni, Caenorhabditis elegans*	in vitro	[34]
Curcuma longa (Stem, leaf and root)	*Ascaridia galli in* Chicken model	in vitro	[35]
Plumbago indica	*Caenorhabditis elegans*	in vitro	[34]
Piper nigrum	*Schistosoma mansoni Caenorhabditis elegans*	in vitro	[34]
Piper chaba	*Schistosoma mansoni Caenorhabditis elegans*	in vitro	[34]
Hibiscus esculentus	*Schistosoma mansoni*	in vitro	[34]
Cordyline fruticosa	*Schistosoma mansoni*	in vitro	[34]
Michelia champaca	*Schistosoma mansoni*	in vitro	[34]
Butea monosperma	*Schistosoma mansoni*	in vitro	[34]
Phyllanthus emblica	*Caenorhabditis elegans*	in vitro	[34]
Baccaurea ramiflora	*Schistosoma mansoni*	in vitro	[34]
Cassia fistula	*Caenorhabditis elegans*	in vitro	[34]
Diospyros mollis	*Caenorhabditis elegans*	in vitro	[34]
Agerantum conyzoides	*Schistosoma mansoni*	in vitro	[34]
Averrhoa carambola	*Schistosoma mansoni*	in vitro	[34]
Plumeria acuminate	*Schistosoma mansoni*	in vitro	[34]
Bouea burmanica	*Schistosoma mansoni*	in vitro	[34]
Spephamia rotunda	*Schistosoma mansoni*	in vitro	[34]
Areca Catechu	*Schistosoma mansoni, Caenorhabditis elegans*	in vitro	[34]
Cocos nucifera	*Haemonchus* contortus- and Sheep model with gastro intestinal parasites	in vitro and in vivo	[36]
Terminalia arjuna	*Haemonchus* contortus	in vitro	[37]
Dioscorea bulbifera	*Eisenia foetida, Raillietina spiralis, Ascardia galli*	in vitro	[38]

Cassia auriculata	*Eisenia foetida, Raillietina spiralis, Ascardia galli*	in vitro	[38]
Erythrina variegata	*Eisenia foetida, Raillietina spiralis, Ascardia galli*	in vitro	[38]
Euphorbia thymifolia	*Pheritima posthuma Ascaridia galli/*	in vitro	[39]
Cassia tora	*Pheritima posthuma Ascaridia galli/*	in vitro	[40]
Ficus obtusifolia	*Toxocara canis*	in vitro	[41]
Acokanthera oppositifolia	*Caenorhabditis elegans*	in vitro	[42]
Felicia erigeroides	*Caenorhabditis elegans*	in vitro	[42]
Cotyledon orbiculata var. dactylopsis	*Caenorhabditis elegans*	in vitro	[42]
Cotyledon orbiculata var. otbiculata	*Caenorhabditis elegans*	in vitro	[42]
Cyathea dregei	*Caenorhabditis elegans*	in vitro	[42]
Senna petersiana	*Caenorhabditis elegans*	in vitro	[42]
Hypoxis hemerocallidea	*Caenorhabditis elegans*	in vitro	[42]
Hypoxis colchicifolia	*Caenorhabditis elegans*	in vitro	[42]
Clerodendrum myricoides (leaf and stem)	*Caenorhabditis elegans*	in vitro	[42]
Camellia sinensis	*Pheritima posthuma*	in vitro	[43]
Clerodendrum myricoides (root)	*Pheretima posthuma*	in vitro	[44]
Ocimum basilicum (Leaf)	*Caenorhabditis elegans*	in vitro	[42]
Ocimum basilicum (leaf, stem and root)	*Pheritima posthuma*	in vitro	[45]
Alocasia indica (Leaves)	*Pheretima posthuma*	in vitro	[46]
Alocasia indica Schott. (rootstocks)	*Pheretima posthuma*	in vitro	[47]
Aloe ferox	*Haemonchus contortus*	in vitro	[48]
Elephantorrhiza elephantina	*Haemonchus contortus*	in vitro	[48]
Leonotis leonurus	*Haemonchus contortus*	in vitro	[48]
Hyptis Suaveolens	*Pheretima posthuma Ascaridia galli*	in vitro	[49]
Passiflora edulis	*Eisenia fetida*	in vitro	[50]
Leucosidea sericea	*Caenorhabditis elegans*	in vitro	[42]
Plumbago zeylanica	*Pheretima posthuma*	in vitro	[51]
Trianthema portulacastrum	*Haemonchus contortus*	in vitro	[52]

Musa paradisiaca	*Haemonchus contortus*	in vitro	[52]
Ficus carica	*Caenorhabditis elegans*	in vitro	[53]
Ormosia fordiana	*Caenorhabditis elegans*	in vitro	[53]
Tsoongiodendron odorum	*Caenorhabditis elegans*	in vitro	[53]
Manglietia aromatica	*Caenorhabditis elegans*	in vitro	[53]
Altingia yunnanensis	*Caenorhabditis elegans*	in vitro	[53]
Elaeocarpus decipiens	*Caenorhabditis elegans*	in vitro	[53]
Momordica cochinchinensis	*Caenorhabditis elegans*	in vitro	[53]
Pistacia weinmannifolia	*Caenorhabditis elegans*	in vitro	[53]
Catharanthus roseus	*Caenorhabditis elegans*	in vitro	[30]
Eclipta prostrata	*Caenorhabditis elegans*	in vitro	[30]
Solanum torvum	*Caenorhabditis elegans*	in vitro	[30]
Annona squamosa	*Caenorhabditis elegans*	in vitro	[30]
Khaya senegalensis (leaf, bark)	*Onchocerca ochengi, Caenorhabditis elegans*	in vitro	[15]
Khaya senegalensis (bark)	*Echinostoma caproni Schistosoma mansoni Heligmosomoides bakeri Trichuris muri*	in vitro	[14]
Annona senegalensis	*Onchocerca ochengi Caenorhabditis elegans*	in vitro	[15]
Parquetina nigrescens	*Onchocerca ochengi Caenorhabditis elegans*	in vitro	[15]
Ficus exasperata	*Onchocerca ochengi, Caenorhabditis elegans*	in vitro	[15]
Euphorbia hirta	*Onchocerca ochengi Caenorhabditis elegans*	in vitro	[15]
Markhamia obtusifolia	*Trichostrongylus colubriformis*	in vitro	[54]
Brassica nigra	*Pheritima posthuma*	in vitro	[45]
Rumex abyssinicus (Root)	*Pheritima posthuma*	in vitro	[45]
Rumex abyssinicus (Leaf and stem)	*Haemonchus contortus*	in vitro	[55]
Cassia alata	*Haemonchus contortus*	in vitro	[56]

Clitoria ternatea	*Eisenia foetida, Raillietina spiralis, Ascardia galli*	in vitro	[57]
Senna occidentalis	*Haemonchus contortus*	in vitro	[55]
Leucas martinicensis	*Haemonchus contortus*	in vitro	[55]
Leonotis ocymifolia	*Haemonchus contortus*	in vitro	[55]
Albizia schimperiana	*Haemonchus contortus*	in vitro	[55]
Linospora Cordifolia	*Eisenia foetida*	in vitro	[58]
Agave sisalana	Gastrointestinal nematodes of goat	in vivo and in vitro	[59-62]
Lannea barteri	*Echinostoma caproni Schistosoma mansoni Heligmosomoides bakeri Trichuris muri*	in vitro	[14]
Sclerocarya birrea	*Echinostoma caproni Schistosoma mansoni Heligmosomoides bakeri Trichuris muri*	in vitro	[14]
Xylopia aethiopica	*Echinostoma caproni Schistosoma mansoni Heligmosomoides bakeri Trichuris muri*	in vitro	[14]
Parinari curatellifolia	*Echinostoma caproni Schistosoma mansoni Heligmosomoides bakeri Trichuris muri*	in vitro	[14]
Parinari excelsa	*Echinostoma caproni Schistosoma mansoni Heligmosomoides bakeri Trichuris muri*	in vitro	[14]
Anogeissus leiocarpus	*Echinostoma caproni Schistosoma mansoni Heligmosomoides bakeri Trichuris muri*	in vitro	[14]
Combretum mucronatum	*Echinostoma caproni Schistosoma mansoni Heligmosomoides bakeri Trichuris muri*	in vitro	[14]
Terminalia avicennioides	*Echinostoma caproni Schistosoma mansoni Heligmosomoides bakeri Trichuris muri*	in vitro	[14]

Monotes kerstingii	*Echinostoma caproni Schistosoma mansoni Heligmosomoides bakeri Trichuris muri*	in vitro	[14]
Anthostema senegalenseis	*Echinostoma caproni Schistosoma mansoni Heligmosomoides bakeri Trichuris muri*	in vitro	[14]
Milbraedia paniculata	*Echinostoma caproni Schistosoma mansoni Heligmosomoides bakeri Trichuris muri*	in vitro	[14]
Eriosema griseum	*Echinostoma caproni Schistosoma mansoni Heligmosomoides bakeri Trichuris muri*	in vitro	[14]
Erythrina senegalensis	*Echinostoma caproni Schistosoma mansoni Heligmosomoides bakeri Trichuris muri*	in vitro	[14]
Piliostigma thonningii	*Echinostoma caproni Schistosoma mansoni Heligmosomoides bakeri Trichuris muri*	in vitro	[14]
Sacoglottis gabonensis	*Echinostoma caproni Schistosoma mansoni Heligmosomoides bakeri Trichuris muri*	in vitro	[14]
Napoleona vogelii	*Echinostoma caproni Schistosoma mansoni Heligmosomoides bakeri Trichuris muri*	in vitro	[14]
Flabellaria paniculata	*Echinostoma caproni Schistosoma mansoni Heligmosomoides bakeri Trichuris muri*	in vitro	[14]
Pseudocedrela kotsstchyi	*Echinostoma caproni Schistosoma mansoni Heligmosomoides bakeri Trichuris muri*	in vitro	[14]

Olax subscorpioidea	*Echinostoma caproni Schistosoma mansoni Heligmosomoides bakeri Trichuris muri*	in vitro	[14]
Securidaca longepedunculata	*Echinostoma caproni Schistosoma mansoni Heligmosomoides bakeri Trichuris muri* Mouse model - *Heligmosomoides polygyrus*	in vitro and in vivo	[14,63]
Craterispermum caudatum	*Echinostoma caproni Schistosoma mansoni Heligmosomoides bakeri Trichuris muri*	in vitro	[14]
Crossopteryx febrifuga	*Echinostoma caproni Schistosoma mansoni Heligmosomoides bakeri Trichuris muri*	in vitro	[14]
Morinda longiflora	*Echinostoma caproni Schistosoma mansoni Heligmosomoides bakeri Trichuris muri*	in vitro	[14]
Sarcocephalus latifoliaus	*Echinostoma caproni Schistosoma mansoni Heligmosomoides bakeri Trichuris muri*	in vitro	[14]
Mimusops kummel	*Echinostoma caproni Schistosoma mansoni Heligmosomoides bakeri Trichuris muri*	in vitro	[14]
Starchytarpheta cayennensis	*Echinostoma caproni Schistosoma mansoni Heligmosomoides bakeri Trichuris muri*	in vitro	[14]
Leonurus sibiricus	*Pheritima posthuma*	in vitro	[14]
Vitex Negundo	*Pheritima posthuma*	in vitro	[64]
Piper betle Linn	*Pheritima posthuma*	in vitro	[65]
Euphorbia helioscopia (leaf, stem and root)	Sheep - Gastrointestinal nematodes *Haemonchus contortus*	in vitro and in vivo	[66,67]
Clerodendrum colebrookianum	Rat - *Hymenolepis diminuta*	in vivo	[68]

Phytolacca icosandra	*Haemonchus contortus*	in vivo	[69]
Asystasia gangeticum	*Pheritima posthuma Ascaridia galli*	in vitro	[70]
Calamus Leptospadix (Seed)	*Pheritima posthuma*	in vitro	[71]
Parthenium hysterophorus	*Pheritima posthuma*	in vitro	[72]
Epiprinus mallotiformis	*Pheritima posthuma*	in vitro	[73]
Robinia pseudoacacia	*Caenorhabditis elegans*	in vitro	[74]
Quercus alba	*Caenorhabditis elegans*	in vitro	[74]
Lespedeza cuneata	*Caenorhabditis elegans*	in vitro	[74]
Salix X sepulcralis	*Caenorhabditis elegans*	in vitro	[74]
Rosa multiflora	*Caenorhabditis elegans*	in vitro	[74]
Acer rubrum	*Caenorhabditis elegans*	in vitro	[74]
Rhus typhina	*Caenorhabditis elegans*	in vitro	[74]
Flacourtia sepiaria	*Pheritima posthuma*	in vitro	[75]
Cymbopogo citratus	*Pheritima posthuma*	in vitro	[76]
Polygonum lapathifolium	*Pheritima posthuma*	in vitro	[77]
Zingiber officinale	*Ascaridia galli in* Chicken model	in vivo	[35]
Avicennia germinans	*Haemonchus contortus*	in vitro	[78]
Lysiloma latisiliquum	*Haemonchus contortus*	in vitro	[78]
Theobroma cacao	*Haemonchus contortus*	in vitro	[78]
Rhizophora mangle	*Haemonchus contortus*	in vitro	[78]
Laguncularia racemosa	*Haemonchus contortus*	in vitro	[78]
Linariantha bicolor	*Caenorhabditis elegans*	in vitro	[79]
Lansium domesticum	*Caenorhabditis elegans*	in vitro	[79]
Picria fel-terrae	*Caenorhabditis elegans*	in vitro	[79]
Rhus aromatica	*Haemonchus contortus*	in vitro	[80]
Perideridia gairdneri	*Haemonchus contortus*	in vitro	[80]
Chrysothamnus viscidiflora	*Haemonchus contortus*	in vitro	[80]
Liatris punctata	*Haemonchus contortus*	in vitro	[80]
Ericameria nauseosa	*Haemonchus contortus*	in vitro	[80]
Melilotus alba	*Haemonchus contortus*	in vitro	[80]
Melilotus officinalis	*Haemonchus contortus*	in vitro	[80]

Geranium viscosissium	*Haemonchus contortus*	in vitro	[80]
Enhydra fluctuans	*Pheritima posthuma*	in vitro	[81]
Spilanthes calva	*Ascaridia galli*<br>*Pheritima posthuma*	in vitro	[82]
Heliotropium	*Pheritima posthuma*	in vitro	[83]
Momordica charantia	*Caenorhabditis elegans*	in vitro	[84]
Eucalyptus globulus	Sheep - Gastrointestinal nematodes	in vivo	[85]
Terminalia citrina	*Pheretima posthuma*	in vitro	[86]
Anacardium occidentale	*Ascaridia galli*	in vitro	[87]
Allium sativum	*Ascaridia galli*	in vitro	[87]
Tribulus terrestris,	*Ascaridia galli*	in vitro	[87]
Bassia latifolia	*Ascaridia galli*	in vitro	[87]
Piper betle	*Ascaridia galli*	in vitro	[87]
Morinda citrifolia L	*Ascaridia galli*	in vitro	[87]
Aloe secundiflora	*Ascaridia galli*	in vitro	[87]
Cassia occidentalis L	*Ascaridia galli*	in vitro	[87]
Psorelia corylifolia	*Ascaridia galli* in birds	in vivo	[87]
Piper betle	*Ascaridia galli* in birds	in vivo	[87]
Pilostigma thonningi	*Ascaridia galli* in birds	in vivo	[87]
Caesalpinia crista	*Ascaridia galli* in birds	in vivo	[87]
Ocimum gratissimum	*Ascaridia galli* in birds	in vivo	[87]
Anacardium occidentale	*Ascaridia galli* in birds	in vivo	[87]
Moringa oleifera	*Haemonchus contortus*	in vitro	[88]
Alangium. salviifolium,	*Caenorhabditis elegans*	in vitro	[88]
Antidesma. bunius,	*Caenorhabditis elegans*	in vitro	[88]
Aporosa octandra,	*Caenorhabditis elegans*	in vitro	[88]
Barringtonia acutangula,	*Caenorhabditis elegans*	in vitro	[88]
Casearia graveolens	*Caenorhabditis elegans*	in vitro	[88]
Crotalaria. pallida	*Caenorhabditis elegans*	in vitro	[88]
Gmelina arborea,	*Caenorhabditis elegans*	in vitro	[88]
Holarrhera pubescens	*Caenorhabditis elegans*	in vitro	[88]
Hypericum gaitii	*Caenorhabditis elegans*	in vitro	[88]

Lannea coromandelica	*Caenorhabditis elegans*	in vitro	[88]
Macaranga peltata	*Caenorhabditis elegans*	in vitro	[88]
Melastoma malabathricum	*Caenorhabditis elegans*	in vitro	[88]
Millettia extensa	*Caenorhabditis elegans*	in vitro	[88]
Nyctanthes arbor-tristis	*Caenorhabditis elegans*	in vitro	[88]
Proteum. serratum	*Caenorhabditis elegans*	in vitro	[88]
Pterospermum acerifolium	*Caenorhabditis elegans*	in vitro	[88]
Rubus ellipticus	*Caenorhabditis elegans*	in vitro	[88]
Suregada multiflora	*Caenorhabditis elegans*	in vitro	[88]
Symplocos cochinchinensis	*Caenorhabditis elegans*	in vitro	[88]
Ventilago maderaspatana	*Caenorhabditis elegans*	in vitro	[88]
Wrightia arborea	*Caenorhabditis elegans*	in vitro	[88]
Annona squamosa	*Pheretima posthuma*	in vitro	[89]
Azadirachta Indica	*Pheretima posthuma*	in vitro	[89]
Punica granatum	*Pheretima posthuma*	in vitro	[89]
Nepeta cadmea	*Tubifex tubifex*	in vitro	[90]
Artemisia absinthium	*Haemonchus contortus*	in vitro	[91]
Chamomilla recutita	*Haemonchus contortus*	in vitro	[91]
Malva sylvestris	*Haemonchus contortus*	in vitro	[91]
Leea aequata	*Pheretima posthuma*	in vitro	[92]
Ficus insipida latex	Monogeneans	in vitro	[93]
Cryptocarya novoguineensis	*Haemonchus contortus*	in vitro	[94]
Piper methysticum	*Haemonchus contortus*	in vitro	[94]
Cassia occidentalis	Gastrointestinal nematodes of goat	in vitro and in vivo	[95]
Euphorbia hirta	Gastrointestinal nematodes of goat	in vitro and in vivo	[95]

1. McGaw, L.J.; Jäger, A.K.; Van Staden, J. Antibacterial, anthelmintic and anti-amoebic activity in South African medicinal plants. *Journal of Ethnopharmacology* **2000**, *72*, 247-263, doi:10.1016/S0378-8741(00)00269-5.
2. Sparg, S.G.; Van Staden, J.; Jäger, A.K. Efficiency of traditionally used South African plants against schistosomiasis. *Journal of Ethnopharmacology* **2000**, doi:10.1016/S0378-8741(00)00310-X.

3. Fakae, B.B.; Campbell, A.M.; Barrett, J.; Scott, I.M.; Teesdale-Spittle, P.H.; Liebau, E.; Brophy, P.M. Inhibition of glutathione S-transferases (GSTs) from parasitic nematodes by extracts from traditional Nigerian medicinal plants. *Phytotherapy Research* **2000**, *14*, 630-634, doi:10.1002/1099-1573(200012)14:8<630::AID-PTR773>3.0.CO;2-5.
4. Ajaiyeoba, E.O.; Onocha, P.A.; Olarenwaju, O.T. In vitro Anthelmintic Properties of Buchholzia coriaceae and Gynandropsis gynandra Extracts. *Pharmaceutical Biology* **2001**, doi:10.1076/phbi.39.3.217.5936.
5. McGaw, L.J.; Rabe, T.; Sparg, S.G.; Jäger, A.K.; Eloff, J.N.; Van Staden, J. An investigation on the biological activity of Combretum species. *Journal of Ethnopharmacology* **2001**, doi:10.1016/S0378-8741(00)00405-0.
6. Enwerem, N.M.; Okogun, J.I.; Wambebe, C.O.; Okorie, D.A.; Akah, P.A. Anthelmintic activity of the stem bark extracts of Berlina grandiflora and one of its active principles, betulinic acid. *Phytomedicine* **2001**, doi:10.1078/0944-7113-00023.
7. Mølgaard, P.; Nielsen, S.B.; Rasmussen, D.E.; Drummond, R.B.; Makaza, N.; Andreassen, J. Anthelmintic screening of Zimbabwean plants traditionally used against schistosomiasis. *Journal of Ethnopharmacology* **2001**, *74*, 257-264, doi:10.1016/S0378-8741(00)00377-9.
8. Dash, G.K.; Suresh, P.; Sahu, S.K.; Kar, D.M.; Ganapaty, S.; Panda, S.B. Evaluation of Evolvulus alsinoides Linn. for anthelmintic and antimicrobial activities. *Journal of Natural Remedies* **2002**, *2*, 182-185.
9. Githiori, J.B.; Höglund, J.; Waller, P.J.; Baker, R.L. Anthelmintic activity of preparations derived from Myrsine africana and Rapanea melanophloeos against the nematode parasite, Haemonchus contortus, of sheep. *Journal of Ethnopharmacology* **2002**, *80*, 187-191, doi:10.1016/S0378-8741(02)00030-2.
10. Alawa, C.B.I.; Adamu, A.M.; Gefu, J.O.; Ajanusi, O.J.; Abdu, P.A.; Chiezey, N.P.; Alawa, J.N.; Bowman, D.D. In vitro screening of two Nigerian medicinal plants (Vernonia amygdalina and Annona senegalensis) for anthelmintic activity. *Veterinary Parasitology* **2003**, *113*, 73-81, doi:10.1016/S0304-4017(03)00040-2.
11. Ademola, I.O.; Eloff, J.N. Ovicidal and larvicidal activity of Cassia alata leaf acetone extract and fractions on Haemonchus contortus: In vitro studies. *Pharmaceutical Biology* **2011**, doi:10.3109/13880209.2010.526948.
12. Githiori, J.B.; Höglund, J.; Waller, P.J.; Baker, R.L. Evaluation of anthelmintic properties of extracts from some plants used as livestock dewormers by pastoralist and smallholder farmers in Kenya against Heligmosomoides polygyrus infections in mice. *Veterinary Parasitology* **2003**, doi:10.1016/j.vetpar.2003.10.006.
13. Ademola, I.O.; Fagbemi, B.O.; Idowu, S.O. Evaluation of the anthelmintic activity of Khaya senegalensis extract against gastrointestinal nematodes of sheep: In vitro and in vivo studies. *Veterinary Parasitology* **2004**, doi:10.1016/j.vetpar.2004.04.001.

14. Koné, W.M.; Vargas, M.; Keiser, J. Anthelmintic activity of medicinal plants used in Côte d'Ivoire for treating parasitic diseases. *Parasitology Research* **2012**, doi:10.1007/s00436-011-2771-z.
15. Ndjonka, D.; Agyare, C.; Lüersen, K.; Djafsia, B.; Achukwi, D.; Nukenine, E.N.; Hensel, A.; Liebau, E. In vitro activity of Cameroonian and Ghanaian medicinal plants on parasitic (Onchocerca ochengi) and free-living (Caenorhabditis elegans) nematodes. *Journal of Helminthology* **2011**, doi:10.1017/S0022149X10000635.
16. Iqbal, Z.; Lateef, M.; Ashraf, M.; Jabbar, A. Anthelmintic activity of Artemisia brevifolia in sheep. *Journal of Ethnopharmacology* **2004**, doi:10.1016/j.jep.2004.03.046.
17. McGaw, L.J.; Eloff, J.N. Screening of 16 poisonous plants for antibacterial, anthelmintic and cytotoxic activity in vitro. *South African Journal of Botany* **2005**, doi:10.1016/S0254-6299(15)30102-2.
18. Barrau, E.; Fabre, N.; Fouraste, I.; Hoste, H. Effect of bioactive compounds from Sainfoin (Onobrychis viciifolia Scop.) on the in vitro larval migration of Haemonchus contortus: Role of tannins and flavonol glycosides. *Parasitology* **2005**, doi:10.1017/S0031182005008024.
19. Ademola, I.O.; Akanbi, A.I.; Idowu, S.O. Comparative nematocidal activity of chromatographic fractions of Leucaena leucocephala seed against gastrointestinal sheep nematodes. *Pharmaceutical Biology* **2005**, *43*, 599-604, doi:10.1080/13880200500301761.
20. Hymete, A.; Iversen, T.-H.; Rohloff, J.; Erko, B. Screening of Echinops ellenbeckii and Echinops longisetus for biological activities and chemical constituents. *Phytomedicine* **2005**, *12*, 675-679, doi:10.1016/j.phymed.2004.01.013.
21. Wasswa, P.; Olila, D. The in-vitro ascaricidal activity of selected indigenous medicinal plants used in ethno veterinary practices in Uganda. *African Journal of Traditional, Complementary and Alternative Medicines* **2006**, doi:10.4314/ajtcam.v3i2.31150.
22. Bizimenyera, E.S.; Githiori, J.B.; Eloff, J.N.; Swan, G.E. In vitro activity of Peltophorum africanum Sond. (Fabaceae) extracts on the egg hatching and larval development of the parasitic nematode Trichostrongylus colubriformis. *Veterinary Parasitology* **2006**, doi:10.1016/j.vetpar.2006.06.013.
23. Iqbal, Z.; Lateef, M.; Khan, M.N.; Jabbar, A.; Akhtar, M.S. Anthelmintic activity of Swertia chirata against gastrointestinal nematodes of sheep. *Fitoterapia* **2006**, doi:10.1016/j.fitote.2006.05.010.
24. Eguale, T.; Tilahun, G.; Debella, A.; Feleke, A.; Makonnen, E. In vitro and in vivo anthelmintic activity of crude extracts of Coriandrum sativum against Haemonchus contortus. *Journal of Ethnopharmacology* **2007**, doi:10.1016/j.jep.2006.10.003.

25. Hong, L.; Li, G.; Zhou, W.; Wang, X.; Zhang, K. Screening and isolation of a nematicidal sesquiterpene from Magnolia grandiflora L. *Pest Management Science* **2007**, doi:10.1002/ps.1337.
26. Nirmal, S.A.; Malwadkar, G.; Laware, R.B. Anthelmintic activity of Pongamia glabra. *Songklanakarin Journal of Science and Technology* **2007**.
27. McGaw, L.J.; Van der Merwe, D.; Eloff, J.N. In vitro anthelmintic, antibacterial and cytotoxic effects of extracts from plants used in South African ethnoveterinary medicine. *Veterinary Journal* **2007**, *173*, 366-372, doi:10.1016/j.tvjl.2005.09.004.
28. Jabbar, A.; Zaman, M.A.; Iqbal, Z.; Yaseen, M.; Shamim, A. Anthelmintic activity of Chenopodium album (L.) and Caesalpinia crista (L.) against trichostrongylid nematodes of sheep. *Journal of Ethnopharmacology* **2007**, *114*, 86-91, doi:10.1016/j.jep.2007.07.027.
29. Dwivedi, S.; Dwivedi, A.; Kapadia, R.; Kaul, S. Anthelmintic Activity of Alcoholic and Aqueous Extract of Fruits of Terminalia. *Ethnobotanical Leaflets* **2008**, *12*, 741-743.
30. Kamaraj, C.; Rahuman, A.A.; Elango, G.; Bagavan, A.; Zahir, A.A. Anthelmintic activity of botanical extracts against sheep gastrointestinal nematodes, Haemonchus contortus. *Parasitology Research* **2011**, doi:10.1007/s00436-010-2218-y.
31. Costa, C.T.C.; Bevilaqua, C.M.L.; Camurça-Vasconcelos, A.L.F.; Maciel, M.V.; Morais, S.M.; Castro, C.M.S.; Braga, R.R.; Oliveira, L.M.B. In vitro ovicidal and larvicidal activity of Azadirachta indica extracts on Haemonchus contortus. *Small Ruminant Research* **2008**, doi:10.1016/j.smallrumres.2007.09.003.
32. Aswar, M.; Aswar, U.; Watkar, B.; Vyas, M.; Wagh, A.; Gujar, K. Anthelmintic activity of Ficus bengalensis. *International Journal of Green Pharmacy* **2008**, *2*, 170-172.
33. Sawarkar, H.A.; Singh, M.K.; Pandey, A.K.; Biswas, D. In vitro anthelmintic activity of Ficus Benghalensis, Ficus Carica & Ficus Religiosa: A comparative study. *International Journal of Pharmacy and Pharmaceutical Sciences* **2011**, *3*, 152-153.
34. Atjanasuppat, K.; Wongkham, W.; Meepowpan, P.; Kittakoop, P.; Sobhon, P.; Bartlett, A.; Whitfield, P.J. In vitro screening for anthelmintic and antitumour activity of ethnomedicinal plants from Thailand. *Journal of Ethnopharmacology* **2009**, *123*, 475-482, doi:10.1016/j.jep.2009.03.010.
35. Bazh, E.K.A.; El-Bahy, N.M. In vitro and in vivo screening of anthelmintic activity of ginger and curcumin on Ascaridia galli. *Parasitology Research* **2013**, doi:10.1007/s00436-013-3541-x.

36. Oliveira, L.M.B.; Bevilaqua, C.M.L.; Costa, C.T.C.; Macedo, I.T.F.; Barros, R.S.; Rodrigues, A.C.M.; Camurça-Vasconcelos, A.L.F.; Morais, S.M.; Lima, Y.C.; Vieira, L.S.; et al. Anthelmintic activity of Cocos nucifera L. against sheep gastrointestinal nematodes. *Veterinary Parasitology* **2009**, doi:10.1016/j.vetpar.2008.10.018.
37. Bachaya, H.A.; Iqbal, Z.; Khan, M.N.; Sindhu, Z.u.D.; Jabbar, A. Anthelmintic activity of Ziziphus nummularia (bark) and Acacia nilotica (fruit) against Trichostrongylid nematodes of sheep. *Journal of Ethnopharmacology* **2009**, doi:10.1016/j.jep.2009.02.043.
38. Kosalge, S.B.; Fursule, R.A. Investigation of anthelmintic potential of some plants claimed by tribals of satpuda hills. *International Journal of PharmTech Research* **2009**.
39. Taran, M.; Azizi, E.; Shikhvaisi, A.; Asadi, N. The anthelmintic effect of Pistacia khinjuk against protoscoleces of Echinococcus granulosus. *World Journal of Zoology* **2009**, *4*, 291-295.
40. Deore, S.L.; Khadabadi, S.S.; Kamdi, K.S.; Ingle, V.P.; Kawalkar, N.G.; Sawarkar, P.S.; Patil, U.A.; Vyas, A.J. In vitro anthelmintic activity of Cassia tora. *International Journal of ChemTech Research* **2009**.
41. Quesada Romero, L.F.; Castaño Osorio, J.C.; Bilbao, M. Efecto antiparasitario de los extractos etanólicos y etéreos de Ficus obtusifolia Kunth (Moraceae), frente a parásitos de clase nematodos (Toxocara catis y Toxocara canis). *Infectio* **2009**, *13*, 259-267, doi:10.1016/S0123-9392(09)70157-2.
42. Aremu, A.O.; Ndhlala, A.R.; Fawole, O.A.; Light, M.E.; Finnie, J.F.; Van Staden, J. In vitro pharmacological evaluation and phenolic content of ten South African medicinal plants used as anthelmintics. *South African Journal of Botany* **2010**, doi:10.1016/j.sajb.2010.04.009.
43. Dwivedi, G.; Rawal, D.; Nagda, S.; Jain, T. Anthelmintic activity of Tea (Camellia sinensis) extract. *Int J Pharm Sci* **2010**, *1*, 451-453.
44. Das, J.K.; Choudhury, S.; Adhikary, S. Anthelmintic activity of Clerodendrum viscosum. *Oriental Pharmacy and Experimental Medicine* **2011**, doi:10.1007/s13596-011-0021-7.
45. Basha, S.N.; Rekha, R.; Saleh, S.; Yemane, S. Evaluation of Invitro anthelmintic activities of Brassica nigra, Ocimum basilicum and Rumex abyssinicus. *Pharmacognosy Journal* **2011**, doi:10.5530/pj.2011.20.17.
46. Mulla, W.A.; Thorat, V.S.; Patil, R.V.; Burade, K.B. Anthelmintic activity of leaves of Alocasia indica Linn. *International Journal of PharmTech Research* **2010**.

47. Patil, S.H.; Sreenivas, S.A.; Deshmukh, P.V.; Srikanth, M.; Choudhury, A.; Wagh, A.E.; Vijapur, L.S. Anthelmintic activity of Alocasia indica Schott. rootstocks. *International Journal of Drug Development and Research* **2012**.
48. Maphosa, V.; Masika, P.J.; Bizimenyera, E.S.; Eloff, J.N. In-vitro anthelminthic activity of crude aqueous extracts of Aloe ferox, Leonotis leonurus and Elephantorrhiza elephantina against Haemonchus contortus. *Tropical Animal Health and Production* **2010**, *42*, 301-307, doi:10.1007/s11250-009-9421-9.
49. Nayak, P.S.; Nayak, S.; Kar, D.M.; Das, P. In vitro anthelmintic activity of whole plant extracts of Hyptis suaveolens poit. *International Journal of Current Pharmaceutical Research* **2010**.
50. Sangeetha, J.; Soundarya, K.; Santhosh, K.; Sindhura, C. Evaluation of in-vitro anthelmentic property of Passiflora edulis Linn. *Research Journal of Pharmaceutical, Biological and Chemical Sciences* **2010**.
51. Kataki, M.S.; Sharma, D.N.; Kumar, S.; Yadav, R.S.; Rajkumari, A. Antibacterial activity , in vitro antioxidant activity and anthelmintic activity of methanolic extract of Plumbago zeylanica L . leaves. *Journal of Pharmacy Research* **2010**, *3*, 2908 - 2912.
52. Hussain, A.; Khan, M.N.; Iqbal, Z.; Sajid, M.S.; Khan, M.K. Anthelmintic activity of Trianthema portulacastrum L. and Musa paradisiaca L. against gastrointestinal nematodes of sheep. *Veterinary Parasitology* **2011**, doi:10.1016/j.vetpar.2011.02.022.
53. Liu, F.; Yang, Z.; Zheng, X.; Luo, S.; Zhang, K.; Li, G. Nematicidal coumarin from Ficus carica L. *Journal of Asia-Pacific Entomology* **2011**, doi:10.1016/j.aspen.2010.10.006.
54. Nchu, F.; Githiori, J.B.; McGaw, L.J.; Eloff, J.N. Anthelmintic and cytotoxic activities of extracts of Markhamia obtusifolia Sprague (Bignoniaceae). *Veterinary Parasitology* **2011**, doi:10.1016/j.vetpar.2011.06.017.
55. Eguale, T.; Tadesse, D.; Giday, M. In vitro anthelmintic activity of crude extracts of five medicinal plants against egg-hatching and larval development of Haemonchus contortus. *Journal of Ethnopharmacology* **2011**, doi:10.1016/j.jep.2011.04.063.
56. Ademola, I.O.; Eloff, J.N. Anthelminthic activity of acetone extract and fractions of Vernonia amygdalina against Haemonchus contortus eggs and larvae. *Tropical Animal Health and Production* **2011**, *43*, 521-527, doi:10.1007/s11250-010-9727-7.
57. Salhan, M.; Kumar, B.; Tiwari, P.; Sharma, P.; Sandhar, H.K.; Gautam, M. Comparative anthelmintic activity of aqueous and ethanolic leaf extracts of Clitoria ternatea. *International Journal of Drug Development and Research* **2011**.
58. Tiwari, P.; Kumar, B.; Kumar, M.; Kaur, M.; Debnath, J.; Sharma, P. Comparative anthelmintic activity of aqueous and ethanolic stem extract of Iinospora cordifolia. *International Journal of Drug Development and Research* **2011**.

59. Botura, M.B.; Silva, G.D.; Lima, H.G.; Oliveira, J.V.A.; Souza, T.S.; Santos, J.D.G.; Branco, A.; Moreira, E.L.T.; Almeida, M.A.O.; Batatinha, M.J.M. In vivo anthelmintic activity of an aqueous extract from sisal waste (Agave sisalana Perr.) against gastrointestinal nematodes in goats. *Veterinary Parasitology* **2011**, doi:10.1016/j.vetpar.2010.11.039.
60. Botura, M.B.; dos Santos, J.D.G.; da Silva, G.D.; de Lima, H.G.; de Oliveira, J.V.A.; de Almeida, M.A.O.; Batatinha, M.J.M.; Branco, A. In vitro ovicidal and larvicidal activity of Agave sisalana Perr. (sisal) on gastrointestinal nematodes of goats. *Veterinary Parasitology* **2013**, doi:10.1016/j.vetpar.2012.10.012.
61. Domingues, L.F.; Botura, M.B.; Cruz, A.C.F.G.D.; Yuki, C.C.; Silva, G.D.D.; Costa, M.S.; Murphy, G.; Moreira, E.L.T.; Meneses, I.D.S.D.; Almeida, M.D.G.Á.R.D.; et al. Evaluation of anthelmintic activity of liquid waste of Agave sisalana (sisal) in goats. *Revista brasileira de parasitologia veterinaria = Brazilian journal of veterinary parasitology : Orgao Oficial do Colegio Brasileiro de Parasitologia Veterinaria* **2010**, doi:10.1590/S1984-29612010000400018.
62. Silveira, R.X.; Chagas, A.C.S.; Botura, M.B.; Batatinha, M.J.M.; Katiki, L.M.; Carvalho, C.O.; Bevilaqua, C.M.L.; Branco, A.; Machado, E.A.A.; Borges, S.L.; et al. Action of sisal (Agave sisalana, Perrine) extract in the in vitro development of sheep and goat gastrointestinal nematodes. *Experimental Parasitology* **2012**, doi:10.1016/j.exppara.2012.03.018.
63. Adiele, R.C.; Fakae, B.B.; Isuzu, I.U. Anthelmintic activity of Securidaca longepedunculata (Family: Polygalaceae) root extract in mice, in vitro and in vivo. *Asian Pacific Journal of Tropical Medicine* **2013**, doi:10.1016/S1995-7645(13)60150-9.
64. Rastogi, T.; Bhutda, V.; Moon, K.; Aswar, P.; Khadabadi, S. Comparative Studies on Anthelmintic Activity of Moringa Oleifera and Vitex Negundo. *Asian Journal of Research in Chemistry* **2009**.
65. Adate, P.S.; Parmesawaran, S.; Chauhan, Y. In vitro Anthelmintic Activity of Stem Extracts of Piper betle Linn Against Pheritima Posthuma. *Pharmacognosy Journal* **2012**, doi:10.5530/pj.2012.29.10.
66. Lone, B.A.; Chishti, M.Z.; Bhat, F.A.; Tak, H.; Bandh, S.A. In vitro and in vivo anthelmintic activity of Euphorbia helioscopia L. *Veterinary Parasitology* **2012**, doi:10.1016/j.vetpar.2012.04.023.
67. Nalule, A.S.; Mbaria, J.M.; Kimenju, J.W. In vitro anthelmintic potential and phytochemical composition of ethanolic and water crude extracts of Euphorbia heterophylla Linn. *Journal of Medicinal Plants Research* **2013**, doi:10.5897/jmpr12.1174.
68. Yadav, A.K.; Tangpu, V. Anthelmintic activity of ripe fruit extract of Solanum myriacanthum Dunal (Solanaceae) against experimentally induced Hymenolepis diminuta (Cestoda) infections in rats. *Parasitology Research* **2012**, *110*, 1047-1053, doi:10.1007/s00436-011-2596-9.

69. Hernández-Villegas, M.M.; Borges-Argáez, R.; Rodríguez-Vivas, R.I.; Torres-Acosta, J.F.J.; Méndez-González, M.; Cáceres-Farfán, M. In vivo anthelmintic activity of Phytolacca icosandra against Haemonchus contortus in goats. *Veterinary Parasitology* **2012**, doi:10.1016/j.vetpar.2012.04.017.
70. Jiju, V.; Gorantla, M.; Chamundeeswari, D. Evaluation of anthelmintic activity of methanolic extract of Asystasia gangeticum. *Int J Pharm Life Sci* **2013**, *4*, 2727-2730.
71. Chakraborty, D.; Borah, S. In-Vitro Anthelmintic Activity Evaluation of Seed Extract of Calamus leptospadix Griff. *International Journal of Research in Pharmacy & Science* **2014**, *4*.
72. Durga, M.S.S.; Satyavathi, K.; Bhojaraju, P.; Preeti, P.M.; Lovaraju, K.P.; Priya, K.S.; Kumari, Y.R.; Prasad, D.V.; Kanthal, L. Pharmacophore. *Pharmacophore (An International Research Journal)* **2013**, *4*, 275-279.
73. Chandrashekar, M.B.; Naika, R.; Vinayaka, K.S.; Joy Hoskeri, H. Evaluation of anthelmintic and antioxidant activities of Epiprinus mallotiformis leaf extracts. *Asian Journal of Pharmaceutical and Clinical Research* **2013**.
74. Katiki, L.M.; Ferreira, J.F.S.; Gonzalez, J.M.; Zajac, A.M.; Lindsay, D.S.; Chagas, A.C.S.; Amarante, A.F.T. Anthelmintic effect of plant extracts containing condensed and hydrolyzable tannins on Caenorhabditis elegans, and their antioxidant capacity. *Veterinary Parasitology* **2013**, doi:10.1016/j.vetpar.2012.09.030.
75. Sreejith, M.; Kannappan, N.; Santhiagu, A.; Mathew, A.P. Phytochemical, Anti-oxidant and Anthelmintic activities of various leaf extracts of Flacourtia sepiaria Roxb. *Asian Pacific Journal of Tropical Biomedicine* **2013**, *3*, 947-953, doi:10.1016/S2221-1691(13)60184-7.
76. Sherwani, S.K.; Khan, M.M.; Khan, M.U.; Shah, M.A.; Kazmi, S.U. Evaluation of In Vitro anthelmintic activity of Cymbopogon citratus (lemon grass) extract. *International Journal of Pharmacy & Life Sciences* **2013**.
77. Bulbul, L.; Uddin, J.; Sushanta, S.M.; Tanni, S.; Nipa, A.F.; Baul, S. Phytochemial investigation and evaluation of antiemetic & anthelmintic activities of Polygonum lapathifolium roots extract. *International Journal of Pharmacy and Life Sciences* **2013**.
78. Vargas-Magaña, J.J.; Torres-Acosta, J.F.J.; Aguilar-Caballero, A.J.; Sandoval-Castro, C.A.; Hoste, H.; Chan-Pérez, J.I. Anthelmintic activity of acetone-water extracts against Haemonchus contortus eggs: Interactions between tannins and other plant secondary compounds. *Veterinary Parasitology* **2014**, doi:10.1016/j.vetpar.2014.10.008.
79. Kumarasingha, R.; Palombo, E.A.; Bhave, M.; Yeo, T.C.; Lim, D.S.L.; Tu, C.L.; Shaw, J.M.; Boag, P.R. Enhancing a search for traditional medicinal plants with anthelmintic action by using wild type and stress reporter Caenorhabditis elegans strains as screening tools. *International Journal for Parasitology* **2014**, doi:10.1016/j.ijpara.2014.01.008.

80. Acharya, J.; Hildreth, M.B.; Reese, R.N. In vitro screening of forty medicinal plant extracts from the United States Northern Great Plains for anthelmintic activity against Haemonchus contortus. *Veterinary Parasitology* **2014**, *201*, doi:10.1016/j.vetpar.2014.01.008.
81. Kuri, S.; Billah, M.M.; Rana, S.M.M.; Naim, Z.; Islam, M.M.; Hasanuzzaman, M.; Ali, M.R.; Banik, R. Phytochemical and in vitro biological investigations of methanolic extracts of Enhydra fluctuans Lour. *Asian Pacific Journal of Tropical Biomedicine* **2014**, doi:10.12980/APJTB.4.2014C677.
82. Jayaraj, P.; Mathew, B.; Mani, C.; Govindarajan, R. Isolation of chemical constituents from Spilanthes calva DC: Toxicity, anthelmintic efficacy and in silico studies. *Biomedicine and Preventive Nutrition* **2014**, doi:10.1016/j.bionut.2014.04.002.
83. Mahato, K.; Kakoti, B.B.; Borah, S.; Kumar, M. Evaluation of in-vitro anthelmintic activity of Heliotropium indicum Linn. leaves in Indian adult earthworm. *Asian Pacific Journal of Tropical Disease* **2014**, doi:10.1016/S2222-1808(14)60451-5.
84. Vedamurthy, A.B.; Rampurawala, J.; Paarakh, P.M.; Jogaiah, S.; Joy Hoskeri, H. Evaluation of anthelmintic activity of momordica charantia L. Seeds. *Indian Journal of Natural Products and Resources* **2015**, *6*, 153-155.
85. Kanojiya, D.; Shanker, D.; Sudan, V.; Jaiswal, A.K.; Parashar, R. Anthelmintic activity of Ocimum sanctum leaf extract against ovine gastrointestinal nematodes in India. *Research in Veterinary Science* **2015**, doi:10.1016/j.rvsc.2015.01.017.
86. Das, N.; Goshwami, D.; Hasan, M.S.; Raihan, S.Z.; Subedi, N.K. Phytochemical screening and in vitro anthelmintic activity of methanol extract of Terminalia citrina leaves. *Asian Pacific Journal of Tropical Disease* **2015**, doi:10.1016/S2222-1808(15)60881-7.
87. Raza, A.; Muhammad, F.; Bashir, S.; Aslam, B.; Anwar, M.; Naseer, M. In-vitro and in-vivo anthelmintic potential of different medicinal plants against Ascaridia galli infection in poultry birds. *World's Poultry Science Journal* **2016**, *72*, 115-124.
88. Cabardo, D.E.; Portugaliza, H.P. Anthelmintic activity of Moringa oleifera seed aqueous and ethanolic extracts against Haemonchus contortus eggs and third stage larvae. *International Journal of Veterinary Science and Medicine* **2017**, doi:10.1016/j.ijvsm.2017.02.001.
89. Nazneen, F.; Muddassir, M.; Meshram, K.; Umekar, M.; Lohiya, R. Phytochemical Screening and Comparative Anthelmintic Activity of Alchoholic Extracts of Some Herbal Plants. *Journal of Pharmaceutical Sciences and Research* **2017**, *9*, 1240.
90. Kaska, A.; Deniz, N.; Çiçek, M.; Mammadov, R. Evaluation of antioxidant properties, phenolic compounds, anthelmintic, and cytotoxic activities of various extracts isolated from Nepeta cadmea: an endemic plant for Turkey. *Journal of food science* **2018**, *83*, 1552-1559.

91. Váradyová, Z.; Pisarčíková, J.; Babják, M.; Hodges, A.; Mravčáková, D.; Kišidayová, S.; Königová, A.; Vadlejch, J.; Várady, M. Ovicidal and larvicidal activity of extracts from medicinal-plants against Haemonchus contortus. *Experimental parasitology* **2018**, *195*, 71-77.
92. Halder, S.; Saqueeb, N.; Qais, N. Antinociceptive and Anthelmintic Activities of Leaves of Leea aequata. *Dhaka University Journal of Pharmaceutical Sciences* **2018**, *17*, 251-255.
93. Gonzales, A.P.P.F.; Santos, G.G.; Tavares-Dias, M. Anthelminthic potential of the Ficus insipida latex on monogeneans of Colossoma macropomum (Serrasalmidae), a medicinal plant from the Amazon. *Acta parasitologica* **2019**, *64*, 927-931.
94. Herath, H.; Preston, S.; Jabbar, A.; Garcia-Bustos, J.; Addison, R.S.; Hayes, S.; Rali, T.; Wang, T.; Koehler, A.V.; Chang, B.C.H.; et al. Selected alpha-pyrones from the plants Cryptocarya novoguineensis (Lauraceae) and Piper methysticum (Piperaceae) with activity against Haemonchus contortus in vitro. *Int J Parasitol Drugs Drug Resist* **2019**, *9*, 72-79, doi:10.1016/j.ijpddr.2018.12.006.
95. Nsereko, G.; Emudong, P.; Omujal, J.; Acai, J.; Kungu, J.M.; Kabi, F.; Mugerwa, S.; Bugeza, J. Comparison of the efficacy of crude methanolic extracts of Cassia occidentalis and Euphorbia hirta with levamisole-HCL against gastrointestinal nematodes of economic importance to goat production in Uganda. *Tropical animal health and production* **2019**, *51*, 2269-2278.
